# Matrix-free mass spectrometric imaging using laser desorption ionisation Fourier transform ion cyclotron resonance mass spectrometry

**DOI:** 10.1002/rcm.4939

**Published:** 2011-03-14

**Authors:** Richard J A Goodwin, Andrew R Pitt, David Harrison, Stefan K Weidt, Pat R R Langridge-Smith, Michael P Barrett, C Logan Mackay

**Affiliations:** 1 Institute of Infection, Immunity and Inflammation University of GlasgowGlasgow G12 8QQ UK; 2 School of Life & Health Sciences Aston UniversityBirmingham B4 7ET UK; 3 Breakthrough Research Unit and Division of Pathology, Institute of Genetics and Molecular Medicine University of EdinburghEdinburgh EH4 2XR UK; 4 SIRCAMS, School of Chemistry University of EdinburghEdinburgh EH9 3JJ UK

## Abstract

Mass spectrometry imaging (MSI) is a powerful tool in metabolomics and proteomics for the spatial localization and identification of pharmaceuticals, metabolites, lipids, peptides and proteins in biological tissues. However, sample preparation remains a crucial variable in obtaining the most accurate distributions. Common washing steps used to remove salts, and solvent-based matrix application, allow analyte spreading to occur. Solvent-free matrix applications can reduce this risk, but increase the possibility of ionisation bias due to matrix adhesion to tissue sections. We report here the use of matrix-free MSI using laser desorption ionisation performed on a 12 T Fourier transform ion cyclotron resonance (FTICR) mass spectrometer. We used unprocessed tissue with no post-processing following thaw-mounting on matrix-assisted laser desorption ionisation (MALDI) indium-tin oxide (ITO) target plates. The identification and distribution of a range of phospholipids in mouse brain and kidney sections are presented and compared with previously published MALDI time-of-flight (TOF) MSI distributions. Copyright © 2011 John Wiley & Sons, Ltd.

Matrix-assisted laser desorption ionisation mass spectrometry imaging (MALDI MSI) is now employed in many laboratories worldwide for spatially resolving proteins, peptides, lipids and small molecules directly from tissue sections,^[1-6]^ the ability to subsequently display the spatial relative intensities of masses within the mass range collected. To date, the technique has commonly required the use of a MALDI matrix, in an aqueous/solvent solution, applied either as a continuous coating (by manual, mechanical or electrostatic spraying) or as discrete droplets (by manual, mechanical, acoustic or piezoelectric droplet dispensing).^[7-10]^ Recent modifications in matrix application have included the use of solvent-free dry-coating methods.^[11-13]^ Puolitaival *et al.*2008^[11]^ validated this approach by comparison of the distribution of phospholipids obtained using MALDI imaging of mouse brain sections prepared using a dry-coating technique for matrix deposition with those obtained from spray-coated sections. Such an approach can also exclude the ethanol washing/fixation process.

Washing of the tissue section, performed after the tissue has been mounted on the target and before matrix application, is of particular importance for protein and peptide imaging. This step is thought to remove ion-suppressing salts and cell debris, as well as dehydrating the sample and reducing proteolytic activity. The ability to avoid both solvent washing and solvent matrix application steps reduces the risk of target analytes spreading significantly. However, one important variable that is rarely considered when performing MSI is that adhesion/co-crystallisation of matrix may be affected by the tissue surface properties, such as hydrophobicity. This means that variations in detected analyte distributions may result not only from their actual relative abundance in specific tissue regions, but also because of variability in analyte ionisation caused by inconsistencies in the matrix density/adhesion.

Matrix-free laser desorption ionisation (LDI) MS imaging has previously been reported for the detection of secondary metabolites in plant tissues. However, the detected metabolites were low mass UV absorbing molecules.^[14]^ In addition, the use of nano-assisted laser desorption ionisation (NALDI) imaging has recently been reported,^[15]^ in which tissue imprints are made to NALDI plates, by thaw-mounting followed by removal of the tissue section by washing. The NALDI plates, with the lipid imprints, are then analysed by laser desorption ionisation using a 9.4 T Fourier transform ion cyclotron resonance (FTICR) mass spectrometer.

Here we report the use of matrix-free laser desorption ionisation mass spectrometry imaging for the detection of high-mass phospholipids directly from tissue sections with no processing, post-thaw-mounting to a MALDI indium-tin oxide (ITO) target, using a 12 T FTICR mass spectrometer. This approach eliminates any risk of analyte spreading during tissue processing. Identification is achieved by a combination of accurate intact mass measurement, collision-induced dissociation (CID) MS/MS fragmentation, and by comparison of the isotope distributions observed with previously published phospholipid MALDI MSI data. In addition to matrix-free LDI MSI data for the phospholipid distributions in mouse brain sections, matrix-free LDI MSI data is also presented for the phospholipid distribution in mouse kidney sections.

## EXPERIMENTAL

All tissues collected were fresh-frozen. Animals were sacrificed in accordance with the UK Animals (Scientific Procedures) Act, 1986, and local ethical guidelines. After sacrifice, organs were immediately snap-frozen by immersion in dry-ice chilled isopentane, before being stored at −80 °C until required for sectioning. The organs were cut by cryostat microtome into 14 μm thick sagittal sections and thaw mounted onto ITO-coated MALDI slides^[16]^ (Bruker Daltonics, Bremen, Germany; Cat. # 237001). The slides were then freeze-dried for approximately 4 h before being stored at −80 °C. All sections were dried after removal from the freezer using a gentle stream of oxygen-free nitrogen (OFN). Optical images were taken using a mounted CCD digital camera and macro lens prior to analysis.

Mass spectra were acquired on a 12T solariX FTICR mass spectrometer (Bruker Daltonics, Billerica, MA, USA), equipped with a combined electrospray/MALDI source, incorporating a smartbeam-II™ 1 kHz laser. Instrument control was achieved using solariX control version 1.5.0 (build 42.8), Hystar 3.4 (build 8) and FlexImaging 2.1 (build 15). Each analysis was the result of 400 laser shots, using a laser diameter of ~30 μm and a power level of 80%. Ions were detected between *m/z* 200 and 3000, yielding a 1Mword time-domain transient. Imaging data were collected with a raster spacing of 100 μm. The data were analysed using FlexImaging. Ions were selected with a mass precision of ±0.001 *m/z* units. For tandem mass spectrometry experiments, using CID, the mass resolving quadrupole was set to an isolation width of 3 *m/z* units, and a collision voltage of 19 V was used.

## RESULTS AND DISCUSSION

In order to demonstrate the effectiveness of this matrix-free approach for laser desorption ionisation FTICR MSI, phospholipid distributions in mouse brain tissue sections were recorded for comparison with previously published MSI phospholipid distributions recorded using solvent-free dry matrix MALDI TOF MS.^[11]^ In order to demonstrate that imaging mass spectra can be generated for other tissue types, using matrix-free LDI on an FTICR MS instrument, spectra were collected from mouse kidney sections as well as the mouse brain sections.

**Figure 1:**
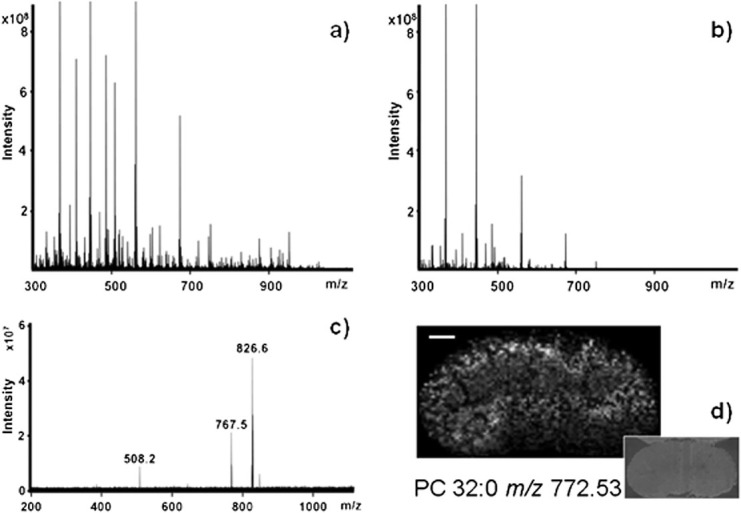
Matrix-free LDI FTICR MSI data: (a) matrix-free LDI spectrum of mouse brain section collected from 200 laser shots, with a laser spot diameter of 30 μm; (b) matrix-free LDI spectrum of mouse kidney section collected from 200 laser shots, with a laser spot diameter of 50 μm; and (c) CID MS/MS spectrum from mouse brain section for the potassium salt adduct of phosphatidylcholine PC (36:2). Annotated are the *m*/z 826.6 precursor ion, as well as the product ion at *m/z* 767.5, resulting from the neutral loss of trimethylamine from the choline head group [M+K−59]^+^. The peak at *m/z* 508.2 corresponds to the loss of one of the side chains (theoretical C_26_H_46_O_5_PK, *m/z* 508.2714). (d) Spatial distribution of PC (32:0), *m/z* 772.5, across mouse kidney section at 100 μm raster. Scale bar 1 mm; insert is an optical image taken with a CCD camera on the macro lens.

Figures 1(a) and 1(b) show matrix-free LDI mass spectra collected from mouse brain and kidney tissue sections, respectively. The tissue sections had not been processed other than by sectioning prior to thaw mounting onto the target plate. Each individual spectrum collected was found to contain several hundred unique features. While the number of mass spectral features is lower in the spectrum for the kidney section, the observed peaks are still of significant abundance. The lower number of features in the spectrum could be the result of ionisation suppression by salts present at higher concentration in the kidney section.

In the present work, unambiguous confirmation of the identity of phospholipid ions was achieved using both the accurate mass of the intact ion, as well as the accurate masses of product ions in the CID tandem mass spectra. For example, accurate mass measurement of the ion at *m/z* 826.6 indicates the species detected at this mass to be the potassium salt of phosphatidylcholine PC (36:1) (theoretical C_44_H_86_NO_8_PK, *m/z* 826.5723).^[11]^

The subsequent CID fragmentation of this precursor ion, see Fig.1(a), clearly shows the neutral loss of trimethylamine from the choline head group peak [M+K−59]^+^ at *m/z* 767.5 (theoretical C_41_H_77_O_8_PK, *m/z* 767.4988).

**Figure 2:**
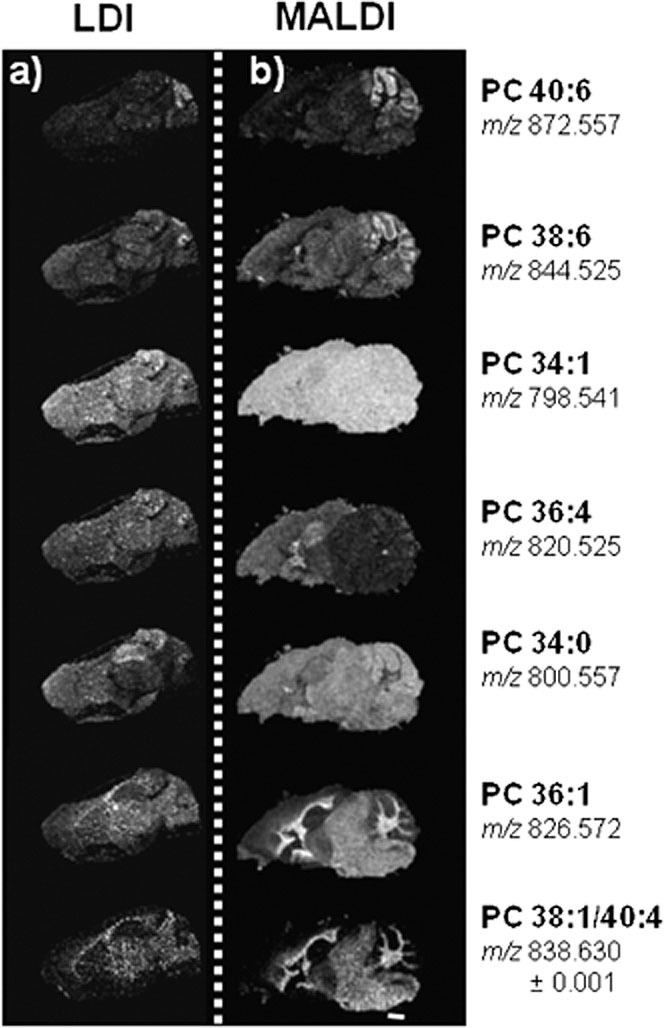
Comparison of matrix-free LDI FTICR mass spectrometry images for phospholipid distributions across mouse brain section, with previously reported distributions obtained using MALDI TOF MSI. Scale bar 1 mm: (a) matrix-free LDI FTICR MSI data, 100 μm raster, mass window filter set to 0.001 *m/z* units; (b) MALDI TOF MSI data using solvent-free dry DHB matrix method, 100 μm raster, MALDI TOF. Reproduced from Puolitaival *et al*.^[11]^

In the earlier study by Puolitaival *et al*.,^[11]^ they compared the phospholipid distributions in mouse brain tissue sections, obtained by MALDI MSI, using a standard solvent-based wet matrix application method with their novel solvent-free dry matrix method. Here we further compare their results with the phospholipid distributions obtained in this work using matrix-free LDI FTICR MSI. Our data was collected using a 30 μm diameter laser spot, at a raster of 100 μm spot-centre to spot-centre. Figure2 shows the LDI FTICR MSI distribution of seven phospholipids in a mouse brain section, which have similar distributions to those detected by MALDI TOF MSI using 2,5-dihydroxybenzoic acid (DHB) matrix applied as a dry coating. As expected, different regions of the tissue appear to have significantly different abundance of phospholipid expression. For example, in both the MALDI TOF MSI and matrix-free LDI FTICR MSI distributions there appears to be a greater abundance of the potassium salt of phosphatidylcholine PC (40:6) (*m/z* 872.557) in the cerebellum. The same is true for PC (38:6), *m/z* 844.525, although the localisation appears less specific. For the remaining lipid distributions there appear to be region-specific distributions, although PC (34:1), *m/z* 798.541, appears to be evenly distributed across the brain sections.

Previously reported MALDI and NALDI MS images of mouse kidney sections have shown the distribution of an ion corresponding to *m/z* 772.5.^[15]^ It was reported that this species was predominantly only present in the renal cortex, and at significantly lower abundance (by NALDI) or absent (by MALDI) in the renal medulla. However, the matrix-free LDI MS images obtained here, using the inherently higher mass measurement accuracy available by FTICR MS, show that the distribution of this ion, *m/z* 772.3, the potassium salt of PC (32:0), is higher in the renal cortex and also present in the renal medulla; see Fig.1(d). The difference in detection efficiency by MALDI or NALDI is presumably a result of the tissue washing and matrix application required prior to analysis.

## CONCLUSIONS

Matrix deposition for MALDI has been a crucial step in the sample preparation protocol for previously reported MSI studies. Matrix deposition technologies have been widely studied, since rapid and reproducible matrix deposition is currently difficult. Each preparation variable, tissue washing, matrix composition or application, results in a complementary profile of detected masses. Each preparation, to a greater or lesser extent, will modify the detected distribution of a given analyte in the tissue. Clearly, the ability to perform matrix-free LDI MSI means that considerably less tissue preparation or modification is required. Therefore, we believe that matrix-free LDI FTICR MS will provide a valuable additional method for tissue imaging, and may accelerate future high-throughput biomedical studies.
